# Unidirectional Magnetic Anisotropy in Dense Vertically-Standing Arrays of Passivated Nickel Nanotubes

**DOI:** 10.3390/nano10122444

**Published:** 2020-12-07

**Authors:** Claudiu Locovei, Nicolae Filipoiu, Andrei Kuncser, Anda-Elena Stanciu, Ştefan Antohe, Camelia-Florina Florica, Andreea Costas, Ionuţ Enculescu, Luc Piraux, Victor Kuncser, Vlad-Andrei Antohe

**Affiliations:** 1 National Institute of Materials Physics (NIMP), Atomiştilor Street 405A, 077125 Măgurele, Romania; claudiu.locovei@infim.ro (C.L.); andrei.kuncser@infim.ro (A.K.); anda.stanciu@infim.ro (A.-E.S.); camelia.florica@infim.ro (C.-F.F.); andreea.costas@infim.ro (A.C.); encu@infim.ro (I.E.); 2 Faculty of Physics, R&D Center for Materials and Electronic & Optoelectronic Devices (MDEO), University of Bucharest, Atomiştilor Street 405, 077125 Măgurele, Romania; filip_n95@yahoo.com (N.F.); santohe@solid.fizica.unibuc.ro (Ş.A.); 3 Academy of Romanian Scientists, Splaiul Independenţei 54, 050094 Bucharest, Romania; 4 Institute of Condensed Matter and Nanosciences (IMCN), Université catholique de Louvain (UCLouvain), Place Croix du Sud 1, B-1348 Louvain-la-Neuve, Belgium; luc.piraux@uclouvain.be

**Keywords:** dense arrays of vertically-aligned heterostructured nickel/nickel oxide (Ni/NiO) nanotubes (NTs), supported anodic aluminum oxide (AAO) nanoporous media, template-assisted electrochemical synthesis, unidirectional anisotropy in quasi one-dimensional (1D) nanostructures, exchange bias field and coercivity of cylindrical ferromagnetic/antiferromagnetic Ni/NiO interfaces, micromagnetic simulations

## Abstract

We report the facile and low-cost preparation as well as detailed characterization of dense arrays of passivated ferromagnetic nickel (Ni) nanotubes (NTs) vertically-supported onto solid Au-coated Si substrates. The proposed fabrication method relies on electrochemical synthesis within the nanopores of a supported anodic aluminum oxide (AAO) template and allows for fine tuning of the NTs ferromagnetic walls just by changing the cathodic reduction potential during the nanostructures’ electrochemical growth. Subsequently, the experimental platform allowed further passivation of the Ni NTs with the formation of ultra-thin antiferromagnetic layers of nickel oxide (NiO). Using adequately adapted magnetic measurements, we afterwards demonstrated that the thickness of the NT walls and of the thin antiferromagneticNiO layer, strongly influences the magnetic behavior of the dense array of exchange-coupled Ni/NiO NTs. The specific magnetic properties of these hybrid ferromagnetic/antiferromagnetic nanosystems were then correlated with the morpho-structural and geometrical parameters of the NTs, as well as ultimately strengthened by additionally-implemented micromagnetic simulations. The effect of the unidirectional anisotropy strongly amplified by the cylindrical geometry of the ferromagnetic/antiferromagnetic interfaces has been investigated with the magnetic field applied both parallel and perpendicular to the NTs axis.

## 1. Introduction

In the last decade, the low-dimensional magnetic nanostructures became a subject of intense research, due to the high market needs for novel and innovative applications in the field of sensing and biosensing [1,2], spintronics [3,4], or magnetic recording and data storage [5,6]. In addition, they are extremely useful in fundamental research, where in many situations multidisciplinary implications are tremendously required to properly address the observed magnetic behavior of such low-dimensional systems. Noteworthy, going down to the size of the nanometer level, new phenomenological aspects have to be taken into account, whereas changing the dimensionality or shape of the nanosized magnetic entities, their specific properties can be largely tuned [7,8,9]. Furthermore, the magnetic specificities of such nanoarchitectures can be modified as well, by introducing either inter-element coupling reactions [10] or interfacial interactions [11]. Regarding the latter approach, of high technological importance are the bilayer systems consisting of a ferromagnetic (F) layer interfaced with an antiferromagnetic (AF) component, as can be often found within spin-valve devices based on either Giant Magneto-Resistance (GMR) [12] or Tunneling Magneto-Resistance (TMR) effects [13]. The main magnetic phenomenon specific to these F/AF systems is the unidirectional anisotropy exhibited in different applications, either by the specific exchange bias field (positive or negative), or by the increased coercive fields of the F layer interfacially-coupled to the AF layer, as compared to a simple free F layer [14,15]. Among the available nanoarchitectures today, the two-dimensional (2D) F/AF bilayer systems are mostly used due to their simple preparation protocols and because they are excellent candidates for spin-valve applications [16]. They have been also mostly studied with respect to the dependence of the unidirectional anisotropy parameters on the magnetic and geometric characteristics of the interfaced F and AF layers [17,18,19,20]. Other zero-dimensional (0D) structures (e.g., core/shell nanoparticles) can be similarly synthesized through cost-effective chemical methods [21], being of large interest for the biomedical community [22]. In contrast, much less studied are actually the quasi-one-dimensional (1D) F/AF configurations with axial cylindrical symmetry and vertical ordering (e.g., nanowires—NWs and nanotubes—NTs), mostly due to the complex and challenging preparation requirements [23]. Although the multi-layered NWs with F/AF interface perpendicular to the NWs axis represents a frequent practical choice for the researchers, the induced unidirectional anisotropy which is proportional to the interface area is relatively low, limiting the potential range of applications [24]. A more convenient approach would be a cylindrical F/AF interface with a high surface, more easily to be studied when replacing the solid NWs with dense arrays of NTs of similar geometrical features [25,26,27]. Besides, compared to the NWs [28,29], the adjustable inner cavity of the NTs represents an additional structural parameter that can be used to further control their magnetic properties [30,31]. On the other hand, the dependence of the unidirectional anisotropy parameters on magnetic and geometric characteristics of the F and AF co-axial layers in such 1D hollow structures has not been systematically considered up to now, remaining an open field of high research interest.

A common procedure for the preparation of large dense arrays of NTs is the electrochemical synthesis within a suitable nanoporous template. For instance, the electrochemical growth of dense arrays of magnetic NTs was repeatedly demonstrated using either track-etched polycarbonate (PC) [25,27] or self-supported anodic aluminum oxide (AAO) [32,33] membranes. However, none of these template-assisted routes are capable of providing an acceptable degree of free-standing vertical ordering of the nanostructures, often required in many applications. Alternatively, the supported AAO template represents a promising nanoporous host for the development of NTs able to remain perpendicular to the substrate after its removal [34].

Among the multitude of F materials available today, the systems based on nickel (Ni) have been intensively studied lately, due to its abundance in nature which makes this material a cheap environmentally-friendly candidate, with excellent catalytic properties and with great potential to be used in energy and data storage applications [35,36,37]. Moreover, if desired, Ni can be easily converted into nickel oxide (NiO) which is an AF material with *Néel* temperature of ∼525 K [38,39]. All these particular features render the Ni/NiO system as representative for F/AF structures, being of interest for applications where the interface properties require a facile tuning by changing the physical and geometrical parameters of both Ni and NiO components [40,41]. In this respect, a large area Ni/NiO interface can be constructed by growing first metallic F Ni NTs within the nanopores of a supported AAO template, and to subsequently allow further partial oxidation of their inner walls, to ultimately generate hybrid heterostructured F/AF Ni/NiO NTs [26]. In this approach, a good Ni/NiO interface quality can be simply achieved due to the similar crystalline structure and lattice parameters of both materials, motivating the frequent choice of this F/AF system for many other core/shell nanoarchitectures, ranging from NWs to nanoparticles [35,42].

The present paper focuses on the experimental evidence and magnetic implications of unidirectional anisotropy in NTs with cylindrical F/AF interfaces. In particular, we report on the facile preparation and specific magnetic behavior of large and dense arrays of Ni NTs with internal ultra-thin NiO concentric layers as a prerequisite of the unidirectional anisotropy. The arrays of 1D F/AF nanoarchitectures have been prepared by an electrochemical synthesis of Ni NTs within supported AAO templates, coupled with their partial passivation under normal room-ambient processing conditions, and subsequently followed eventually by the complete removal of the AAO nanoporous host to allow their further morpho-structural investigations. Noteworthy, magnetic investigations of single F Ni NWs and NTs have been already reported [30,33]. Moreover, in a previous paper [26], we even described similar nanostructures with axial symmetry and hollow inner cavities, where the metallic Ni phase was partially converted into NiO through a well-controlled thermal oxidation process, and we investigated the annealing effects on the magnetic properties of these hybrid nanosystems, however without focusing on specific conditions for inducing unidirectional anisotropy and accompanying exchange bias effects. Nevertheless, our present work represents a complementary study, where we additionally have shown that the magnetic behavior is also influenced by the physical dimensions of the Ni NT walls in conditions of maintaining the same ratio between average wall thickness and inner diameter of the NTs in successive samples obtained through changing the cathodic reduction potential during their electrochemical growth. Even more importantly, we also demonstrated that an ultra-thin NiO internal layer obtained by passive oxidation under ambient processing conditions may be sufficient to control the unidirectional anisotropy of such dense arrays of NTs, investigated with the magnetic field applied along and perpendicular to the NTs axis. The later approach could represent a much cheaper alternative to be used in applications where either macroscopic effects of unidirectional anisotropies or the dipolar interaction through the magnetic array need to be further controlled. Ultimately, by adequately-implemented micromagnetic simulations, we have made a step forward in this work towards a deeper understanding of the connection between the morpho-structural as well as geometrical parameters of the NTs and their specific magnetic properties.

## 2. Materials and Methods

### 2.1. Samples Preparation

**Figure 1:**
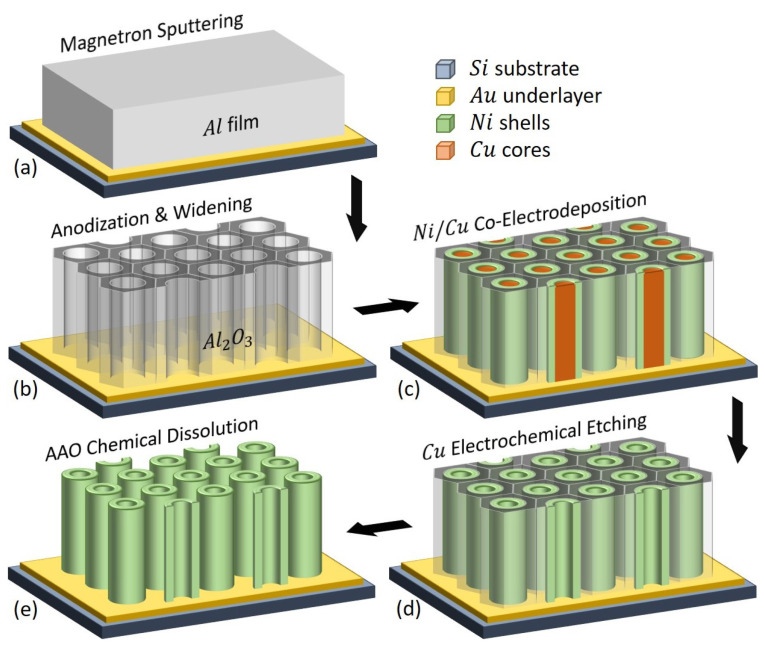
Schematic representation of the vertically-aligned Ni nanotubes (NTs) fabrication process. (**a**) Sputtering of the Au and Al layers on the Si substrate. The ultra-thin adhesion Ti film (as described in the text) was not represented for convenience. (**b**) Formation of the supported nanoporous anodic aluminum oxide (AAO) host through anodization and nanopores widening. (**c**) Cu/Ni core/shell nanowires (NWs) growth by co-electrodeposition (co-ECD). (**d**) Cu cores removal by electrochemical etching. (**e**) Chemical dissolution of the AAO template, revealing the Ni NTs perpendicularly-aligned to the Au/Si substrate.

In this work, analytical grade reagents (Merck) were used as provided. Ultra-pure deionized water (DIW) supplied from a Milli-Q water purification system was used for both preparing the aqueous solutions and rinsing steps of the samples during the fabrication process flow. A pressurized gaseous nitrogen (N_2_) tank (5.0 purity, Linde) was employed as well for cleaning and blow-drying operations. The dense array of parallel vertically-standing magnetic Ni NTs was produced by electrochemical synthesis within a supported nanoporous AAO template (see Figure 1), using an adapted fabrication protocol described elsewhere [26].

In brief, freshly cleaned *p*-doped <100> Si substrates (Siegert Wafer, with a thickness of 525 μm and a resistivity of 0.005 Ω·cm) were first in-situ coated with a multi-layer sequence consisting of Ti/Au/Ti/Al (5/50/5/1000 nm) using a magnetron sputtering equipment (Plassys MP500S), where the Au nobel metal was introduced as a well-conducting electrode withstanding electrochemical conditions, Al layer was used further to develop the supported AAO template through anodization, and the ultra-thin Ti film was employed to enhance the adhesion between the Si substrate and the successive metallic layers of Au and Al, respectively. The plasma was engaged at 15 °C in an Ar atmosphere kept at 5 mTorr, while applying 100 mA DC (to sputter Ti and Au), as well as 1.5 A DC (to sputter Al), for about 2 min and 10 min, respectively (see Figure 1a).

**Figure 2:**
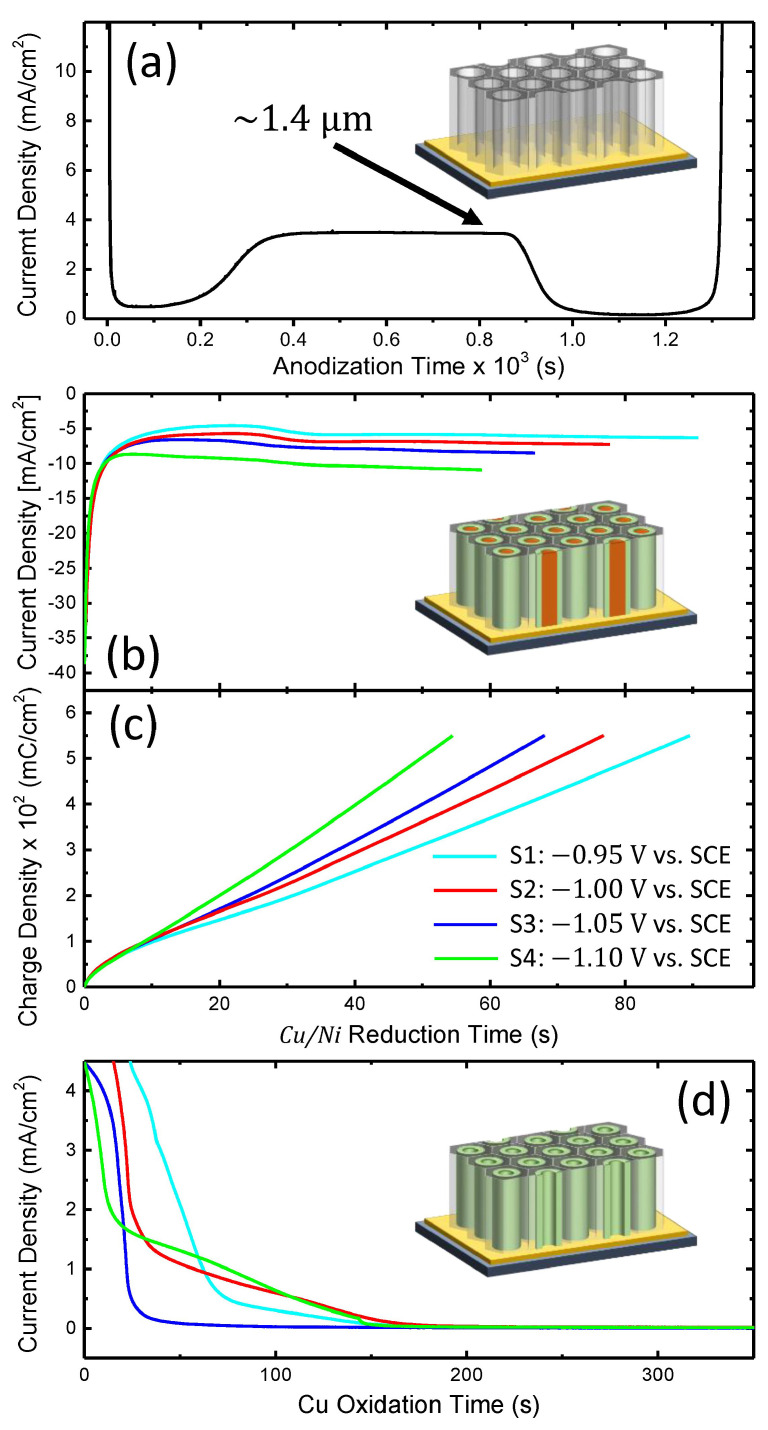
(**a**) Typical current curve obtained for the anodization of the entire Al film, to generate a supported AAO template with a thickness of ∼1400 nm. (**b**) Cathodic reduction current and (**c**) associated charge density obtained during the co-ECD of Cu/Ni core/shell NWs embedded within the supported AAO template, under various potentials applied versus a saturated calomel (SCE) reference electrode. (**d**) Corresponding anodic oxidation current curves for the electrochemical etching of the Cu cores. The insets show schematics of the three equivalent fabrication steps, as detailed in the text.

Afterwards, the as-prepared substrate was installed in a thermoregulated “home-made” electrolytic cell and it was subsequently anodized in a single step to generate the supported AAO template with parallel cylindrical nanochannels down to the Au conducting underlayer (see Figure 1b). The process was carried out in 0.3 M oxalic acid at 2 °C, by applying 60 V from a Keithley 2460 sourcemeter. The obtained anodization current curve is depicted in Figure 2a resembling the normal behavior, where the observed drop followed by a slight increase of the current at the beginning of the process is due to the formation of a barrier-type oxide on the initial Al surface and subsequent nanopores nucleation, while the current plateau is further associated with the nanopores formation through a competitive equilibrium between the Al_2_O_3_ formation and field-enhanced dissolution of the freshly formed Al_2_O_3_ at both metal/metal oxide and metal oxide/electrolyte interfaces, respectively, as reported elsewhere [28,29]. Complementarily, the end of the process is marked by the anodization of the ultra-thin Ti layer pursued by a sharp increase in the current as the nanopores are progressively reaching the Au conducting underlayer. The nanopores were then chemically enlarged in a 0.5 M H_2_SO_4_ solution, at 40 °C for 2 h. The resulting supported AAO template had a thickness of ∼1.4 μm (estimated by integrating the anodization current curve—see Figure 2a, and expected while considering the initially-sputtered Al film thickness of 1 μm) [26].

In a subsequent stage, arrays of Cu/Ni core/shell NWs were grown within the nanopores of the previously constructed supported AAO template (see Figure 1c) by co-electrochemical deposition (co-ECD) at room temperature from an aqueous bath containing: 0.4 M Ni(H_2_NSO_3_)_2_ · 4H_2_O, 0.05 M CuSO_4_ · 5H_2_O and 0.1 M H_3_BO_3_ [25,26,27]. The process was carried out in a “three-electrodes” potentiostatic configuration (using a Voltalab PGZ-301 potentiostat/galvanostat) and the potentials were applied to the working electrode (the substrate) versus a KCl-saturated calomel (SCE) reference electrode (E=0.241 V against Normal Hydrogen Electrode—NHE) with a Pt strip acting as an auxiliary (counter) electrode. The obtained cathodic reduction current curves are depicted in Figure 2b for various applied potentials (−0.95 V—cyan, −1.00 V—red, −1.05 V—blue and −1.1 V—green). As can be noticed in Figure 2b, the AAO-assisted electrochemical growth of the Cu/Ni core/shell NWs is a typical chronoamperometric process involving two stages, i.e., a sharp negative increase of the current in the beginning (corresponding to the formation of a diffusion layer of cations in the solution and the NWs nucleation on the Au cathode at the bottom of the pores), followed by a current plateau until the end of the process (mainly related to the NWs vertical growth within the AAO nanopores when the gradient of ions concentration in the solution reached an equilibrium). It has been reported that the formation and vertical growth of such Cu/Ni core/shell architectures are primarily due to an existing miscibility gap in the solubility of the two metallic species, creating a phase separation with different nucleation rates between the two phases. In this way, the faster Cu reduction induces the initial formation of small Cu islands, while the Ni growth is progressively enhanced at the islands periphery as the solution is depleted of Cu cations [25,26]. Furthermore, it has been also demonstrated that the value of the applied potential could play an essential role in controlling the growth rate ratio between Cu and Ni affecting the thickness of the deposited Cu and Ni layers, respectively, thus the wall thickness of the ultimately obtained Ni NTs [27]. In order to deposit the same volume of materials among the different samples, the total deposition charge was monitored (see Figure 2c), hence depending on the applied potential the growth time was adjusted to obtain for each sample a total charge density of ∼550 mC/cm^2^, corresponding to an average length of the NWs inside the AAO nanopores of ∼400 nm. Next, the Cu cores were electrochemically etched (see Figure 1d) using the same bath and configuration, by applying (versus SCE) a positive potential of +0.2 V. It has been also shown that at small positive potentials Ni does not exhibit a significant etching rate due to its passivation in sulfamate solutions [43,44,45], allowing thus the formation of Ni NTs after the complete Cu electrochemical detachment. Figure 2d shows the anodic oxidation current curves corresponding to the prepared samples, showing a sharp positive increase of the current (due to a large amount of initially-available Cu), followed by its gradual decrease (attributed to a decrease of the oxidation rate due to the progressive reduction of the solid Cu inside the Ni NTs) towards zero once the Cu dissolution ends [46]. In this context, as can be noticed from Figure 2d, a process duration of ∼3 min is enough to entirely secure the Cu cores removal for all samples. Noteworthy, it was previously reported that the template synthesis confinement has an important role in the formation of such Cu/Ni core/shell NWs with easily detachable Cu cores, as the co-ECD within tiny nanopores (with average diameters lower than 50 nm) determines the formation of complex mixed-phase NWs made up of a Cu-Ni alloy [44], as observed also in the case of electrodeposited thin films where the Cu etching step determines the formation of interesting Ni film superficial porous-like morphologies [47].

Finally, the supported AAO host was optionally removed chemically (see Figure 1e) at room temperature, using a 2 M NaOH solution for 15 min, mainly to allow the subsequent morpho-structural analysis of the (passivated) Ni NTs. The samples were afterwards gently rinsed with DIW and then let to dry under atmospheric conditions [34]. In the present study, four samples (S1 to S4) have been prepared in similar conditions, by varying the cathodic reduction potential between −0.95 V and −1.10 V (versus SCE), in order to obtain Ni NTs with slightly different wall thicknesses, as subsequently described. As demonstrated later on, the as-prepared Ni NTs exhibited as well, a stable-in-time thin concentric inner NiO layer, yielded by the surface passivation of the Ni during the electrochemical process flow, eventually coupled with its further passive oxidation under normal room ambient conditions [43,44,45].

### 2.2. Samples Characterization

Morphological observations of the samples were carried out using a *Carl Zeiss* Evo 50 XVP Scanning Electron Microscope (SEM). The quality of the samples was evaluated and the geometrical parameters of the Ni NTs were directly measured. The structural characterization was performed by means of X-ray diffraction (XRD) using a Bruker D8 Discover diffractometer working with a CuK_*α*1_ radiation source ($\lambda_{K_{\alpha 1}}=0.154$ nm). The XRD data were acquired in *Bragg-Brentano* configuration in the range of 2*θ* = 35°–70° and with an angular step of 0.04°. All samples were morphologically and structurally investigated after the electrochemical detachment of the Cu cores and subsequent complete removal of the supported AAO template.

The magnetic measurements were done with a Superconducting Quantum Interference Device (SQUID) magnetometer (Quantum Design MPMS 7T) working under the sensitive Reciprocal Space Option (RSO). Hysteresis loops were acquired at two different temperatures (10 K and 300 K) with the magnetic field applied in two different geometries (parallel and perpendicular to the NTs axis, respectively). Magnetization curves versus temperature were collected at 2000 Oe applied magnetic field, proving a decrease of the specific magnetization of less than 9% when increasing the temperature from 10 K to 300 K (similar to the temperature decrease of the magnetic moment of metallic Ni [48,49]), and hence exhibiting no significant magnetic relaxation. The NT samples (with Cu cores eliminated) were diced into 3×3 mm^2^ rectangular pieces and magnetically characterized without removing the supported AAO template, in order to preserve as much as possible the initial angular orientation of the Ni NTs.

Theoretical magnetic hysteresis loops in the two geometries (with the field applied parallel and perpendicular to the NTs axis) and for different geometrical parameters of the Ni NTs, have been also numerically computed using the Object-Oriented Micromagnetic Framework software package. The driver “Oxs_MinDriver” coupled with the “Oxs_CGEvolve” evolver have been considered for a time-independent approach of the micromagnetic simulations [50].

## 3. Results and Discussion

### 3.1. Morphological and Structural Characterizations

**Figure 3:**
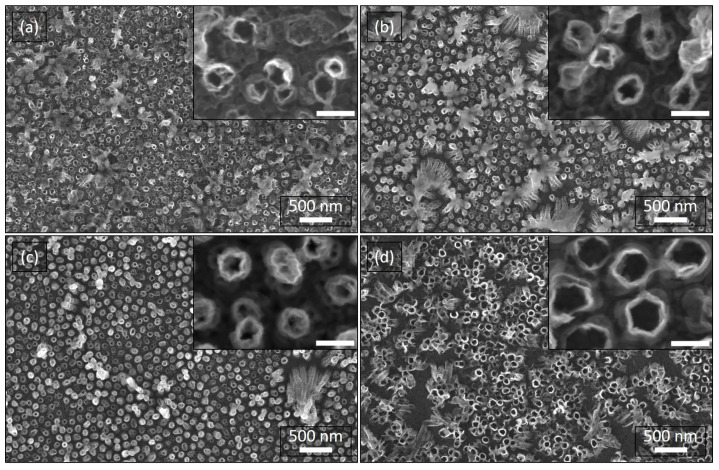
Top-view scanning electron microscope (SEM) micrographs of the dense arrays of Ni NTs vertically-aligned to the Au/Si substrate, prepared under different cathodic reduction potential of the Cu/Ni core/shell NWs (applied versus SCE): (**a**) −0.95 V (sample S1), (**b**) −1.00 V (sample S2), (**c**) −1.05 V (sample S3), and (**d**) −1.10 V (sample S4). The insets represent corresponding higher magnification SEM images (scale bars: 100 nm). The SEM inspections were performed after the electrochemical etching of the Cu cores and subsequent complete removal of the supported AAO template.

Figure 3 shows top-view SEM micrographs (acquired after the complete dissolution of the embedding AAO host) of the dense arrays of vertically-aligned Ni NTs, prepared at different cathodic reduction potentials (applied versus SCE) of the Cu/Ni core/shell NWs (see also Figure 1c and Figure 2b): (a) −0.95 V (sample S1), (b) −1.00 V (sample S2), (c) −1.05 V (sample S3), as well as (d) −1.10 V (sample S4), and subsequent electrochemical etching of the Cu cores at an anodic oxidation potential (applied versus SCE, too) of +0.2 V (see also Figure 1d and Figure 2c). As can be noticed, the obtained Ni NTs retain their predominant perpendicular orientation with respect to the substrate after the AAO matrix removal. Furthermore, a dense homogenous array of Ni NTs can be observed in all situations, with an increasingly better morphological integrity and an increasing wall thickness of the NTs, as the reduction potential is more negative (from S1 to S4). Indeed, the latter remark can be attributed to an increase of the Ni layer with raising the reduction potential [27], hence conferring a better structural quality of the tubular architectures. Consequently, S1 (Figure 3a) exhibits a relatively higher amount of NTs with very thin and irregularly-defined walls, while S2 (Figure 3b) additionally presents specific bunch-like defects (agglomeration and collapsing of several NTs). In contrast, S3 and S4 (Figure 3c,d) manifest a better superficial distribution of regular NTs with a proper dispersion and low concentration of NTs bunches (seen as defects within micromagnetic simulations presented in Section 3.2). However, the overall “curly”-like aspect of the Ni NT walls observed in all samples can be attributed to irregularities during the formation of Cu/Ni core/shell co-axial NWs, with Cu nodules protruding through the Ni layer and leaving-up tracks during the Cu electrochemical etching process [37,47].

**Figure 4:**
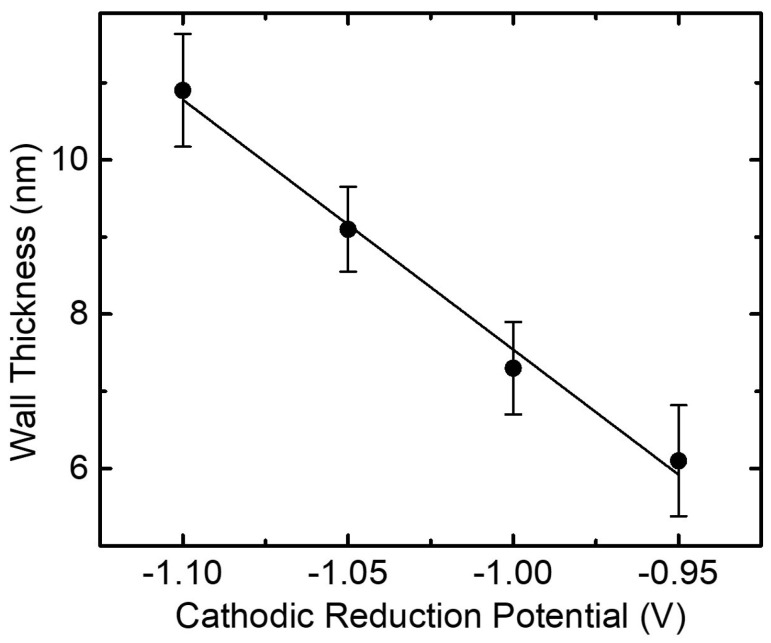
Graphical representation of the NTs average wall thickness dependence as a function of the cathodic reduction potential applied (versus SCE) during the electrochemical growth of Cu/Ni core/shell NWs. The measurements were acquired from the SEM analysis and the error bars represent the standard deviation (n=20).

The geometrical features of the Ni NTs (e.g., wall thickness, inner/outer diameter and center-to-center distance) have been estimated from a series of SEM images taken at various magnifications and in different areas of each sample. A clear linear dependence can be noticed between the mean values of the NTs wall thickness (plotted in Figure 4 with attached error bars) and the cathodic reduction potential applied (versus SCE) during the electrochemical growth of Cu/Ni core/shell NWs (see Figure 1c and Figure 2b), as expected and similarly reported elsewhere [27]. Consequently, the NTs inner diameter was found to vary between 51.5±6.4 nm and 73.1±7.4 nm. However, it can be naturally observed from the SEM images that the values corresponding to both, outer diameter and center-to-center distance, vary differently from one sample to the other too, but the majority of them are within a narrow set of values. The center-to-center distance fluctuating between 87±13.4 nm and 138±12.6 nm can be obviously explained by the different substrates employed for the fabrication of the samples, which although processed in the same conditions, small uncontrollable variations of both substrate preparation characteristics and electrochemical processing parameters, may slightly affect the final product outcome. Noteworthy, all these geometrical parameters extracted from the SEM analysis have been used to magnetically estimate the thickness of both ferromagnetic Ni and antiferromagnetic NiO layers, respectively, as discussed further in Section 3.2.

**Figure 5:**
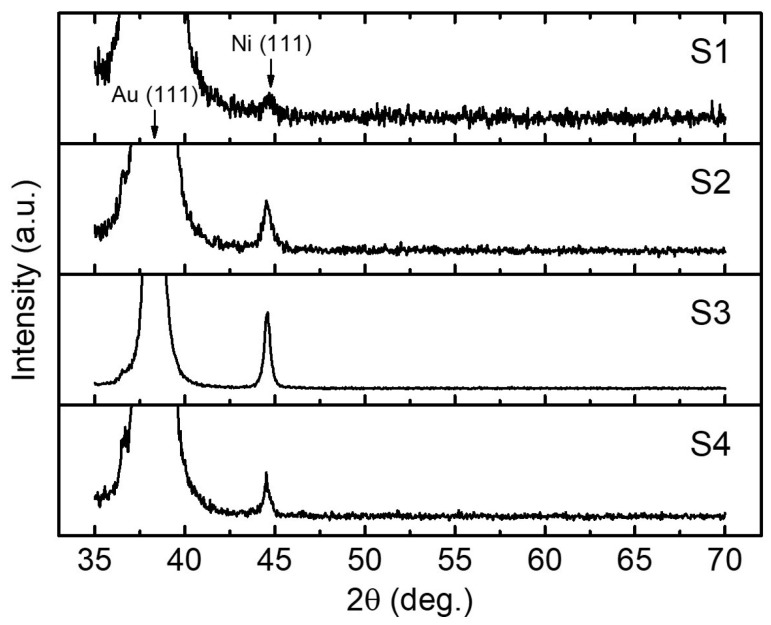
X-ray diffraction (XRD) patterns acquired from the Ni NTs vertically-supported on the Au/Si substrates and prepared by applying (versus SCE) different cathodic reduction potentials for the Cu/Ni core/shell NWs growth: −0.95 V (S1), −1.00 V (S2), −1.05 V (S3) and −1.10 V (S4).

Figure 5 shows the XRD patterns acquired on the samples presented in Figure 3. The typical Au diffraction peaks were observed in all situations being definitely attributed to the Au underlayer on the substrate. Due to the very well defined crystalline structure of the Au film, its only observed preferential orientation is along the (111) reflection planes (JCPDS 04-0784). Furthermore, the metallic Ni (111) crystalline plane is highlighted in all samples (JCPDS 01-1260). This result came not only from the fact that the close-packed (111) plane possesses the lowest surface energy in the *face-centered cubic* structure [51,52], but also from the synthesis parameters together with the preferred orientation of the underlayer, that both play an important role even for large lattice mismatch systems [53,54]. Although the presence of thin NiO layers concentric with the as-prepared Ni NTs is evidenced during the investigation of the magnetic properties (see Section 3.2), noticeable NiO peaks were not present within the XRD analysis, either because the broad Au peaks hinder the visibility of NiO (111) peak around 37° (JCPDS 22-1189), or simply because, during the passive oxidation process of Ni, the Ni cations and oxygen anions had no favorable energies to trigger a proper NiO crystallization. It was shown previously that a well-controlled post-thermal treatment initiates a sintering process that results in a better re-crystallization of both Ni and forming NiO layer [26,27]. Noteworthy, the XRD pattern acquired from S1 shows a disordered structure of NTs, while increasing quality of the crystalline structure can be noticed with increasing the cathodic reduction potential, with very good results obtained for S3. These observations are in good agreement with the previous SEM characterizations of the Ni NTs (see Figure 3) and they are obviously attributed to increasingly better robustness of the NT walls with increasing the cathodic reduction potential. Nevertheless, the slightly noisier XRD spectrum obtained for S4 can be attributed to the strongly negative cathodic potential (−1.1 V versus SCE), where according to Z. Liu et al. [44], the mobilities of the two Ni and Cu cationic species increase differently and the immiscibility separation between them reduces, contributing to the formation of a Cu-rich Ni shell of the Cu/Ni co-axial architecture, that could affect further the crystalline quality of the NTs resulted after the electrochemical detachment of the Cu cores.

### 3.2. Magnetic Properties and Micromagnetic Simulations

**Figure 6:**
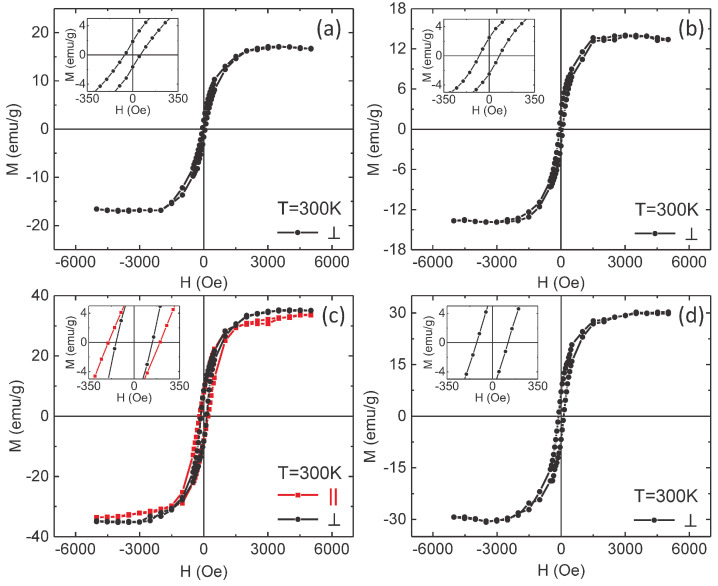
Hysteresis loops of samples S1 (**a**), S2 (**b**), S3 (**c**) and S4 (**d**), recorded at 300 K with the magnetic field applied perpendicular (black lines with dots) and parallel (red line with squares) to the NTs axis. The latter is shown only for S3. The insets represent corresponding closed-up magnifications around the coordinate system’s origin, showing the magnetic coercivity within the prepared samples.

Figure 6 presents typical magnetic hysteresis loops measured by SQUID magnetometry at a temperature of 300 K, with the field applied perpendicular (black line with dots) to the NTs axis. In addition, for S3 (Figure 6c) the hysteresis loop with the field applied parallel to the NTs axis (parallel geometry) is represented as well (red line with squares). In all samples, an almost complete saturation can be seen in perpendicular geometry, reached under saturation fields of about 2000 Oe. Although at a first view the saturation magnetization seems to vary independently on the NTs wall thickness, the subsequent analysis will be done under the assumption of a consistent trend with the real amount of the F phase. As described further, the experimental values of the saturation magnetization per unit mass of magnetic material in these samples are consistently lower than the spontaneous magnetization of metallic Ni. The formation of a passivated NiO internal layer might represent a possible explanation for this behavior, but stronger arguments in this respect are required. A simple way to sustain this hypothesis is by pointing out unreasonable differences between the experimentally obtained hysteresis loops and the theoretical loops obtained via micromagnetic simulations performed on Ni NTs with geometrical parameters close to the experimental ones. One of the main hysteretic parameters to be compared is the coercive field. According to Figure 6, the experimental coercive fields are in the range from 20 Oe to 140 Oe, depending on the sample, with almost similar highest values in samples S3 and S4 (Figure 6c,d). The hysteresis loops in parallel geometry do not differ substantially from the ones in perpendicular geometry, except the slightly higher coercive fields (i.e., ranging from 30 Oe to 200 Oe, depending on the sample), as illustrated for sample S3 in Figure 6c. It is worth noticing that in previous studies on Ni and Ni-Cu NWs, it has been proven that given the strong uniaxial anisotropy along the NW axis, a coherent rotation of the Ni magnetic moment is expected if the field is applied perpendicular to the NW. As a direct consequence, a linear and reversible magnetization reversal between the two (negative and positive) saturation fields is specific to the corresponding hysteresis loops. If the same effect of uniaxial anisotropy is taken into account, the hysteresis loop of an NW in parallel geometry should be rectangular for both coherent rotation of the magnetic moments inside the NW and magnetization reversal through rapid displacements of domain walls [55,56].

**Figure 7:**
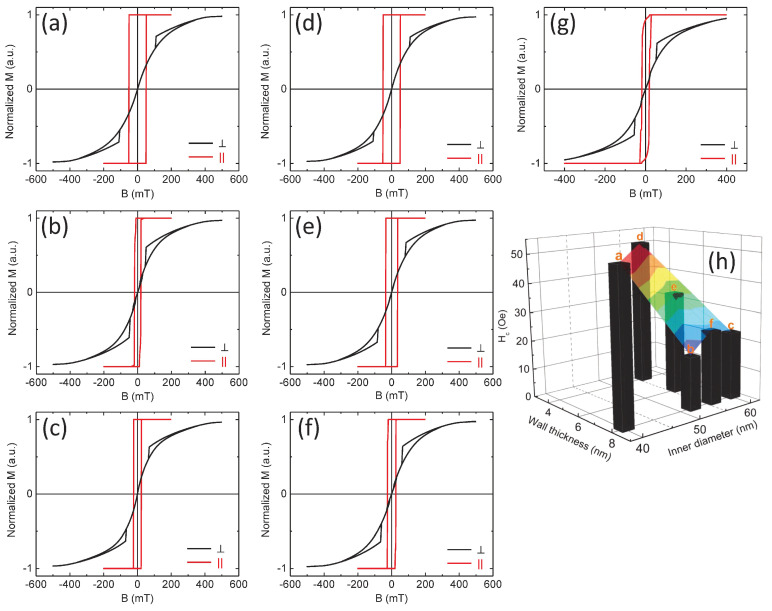
Simulated hysteresis loops of Ni NTs in parallel (red) and perpendicular (black) geometry obtained for the following geometrical parameters: 3 different inner diameters of 40 nm (**a**), 52 nm (**b**) and 60 nm (**c**) for a constant 8 nm wall thickness, as well as three different wall thicknesses of 4 nm (**d**), 6 nm (**e**) and 8 nm (**f**) for the same inner diameter of 56 nm. (**g**) Hysteresis loops for a system of 4 Ni NTs with a wall thickness of 14 nm, an inner diameter of 66 nm and center-to-center distance of 200 nm. (**h**) 3D representation of the theoretical $H_{c}$ as a function of wall thickness and inner diameter. The color of 3D surface representation suggests the highest (red) and lowest (blue) values of $H_{c}$.

Micromagnetic simulations have been performed in order to find out the specific hysteresis loops of the Ni NTs with geometrical characteristics close to the experimental ones. These will be finally discussed in comparison to the experimental hysteresis loops obtained at both 300 K and 10 K. The mesh of the working space has been defined in order to keep the maximum angle between neighboring magnetic moments lower than 30°. Consequently, a 2×2×2 nm cell has been used for the external magnetic field applied along the NT axis, while a 2×2×3 nm cell has been used for the magnetic field applied orthogonal to the NT axis. An aspect ratio (length/radius) of 10 has been used for all simulations, in order to mimic the specific shape anisotropy of a very long NT. Typical magnetic parameters for metallic Ni have been used [57]: spontaneous magnetization $M_{s}\;=\;4.9\times 10^{5}$ A/m, stiffness constant $A=9\times 10^{-12}$ J/m and magnetocrystalline anisotropy coefficient $K=-5.7\times 10^{3}$ J/m^3^ (to note that the computed shape anisotropy energy is highly dominant over the magnetocrystalline one). The evolution of the magnetization reversal has been computed for two situations: (i) NTs with constant diameter and various wall thickness in the range from 4 to 10 nm and (ii) NTs with constant wall thickness and various inner diameters from 40 to 66 nm. Hysteresis loops in the two geometries (parallel and perpendicular) are shown in Figure 7, where a 3D representation of all the coercive fields obtained in parallel geometry is also presented in Figure 7h. According to these results, the hysteresis loops of Ni NTs with geometrical parameters close to the experimental ones do not present coercive field in perpendicular geometry and have rectangular shape in parallel geometry. Another important observation is related to the general trend of the coercive field in parallel geometry: it decreases almost linearly with the wall thickness and even more rapidly with the diameter.

The highest coercive fields obtained via micromagnetic simulations for the lowest diameters are around 50 Oe, but this value corresponds to very thin walls of 4 nm. For wall thicknesses in the range of 6 to 11 nm and inner diameters of 50 to 73 nm, values much lower than 20 Oe are deduced from micromagnetic simulations, namely up to one order of magnitude lower than the maximum experimental coercive field at 300 K. To note that, the used time-independent approach for calculation provides results in the magnetic frozen regime specific to low-temperature measurements. Expectedly, the coercive field at low temperature must be even higher than at 300 K (as confirmed also by the subsequent low-temperature measurements) and so, the discrepancy between the theoretically estimated coercive fields of Ni NTs and the experimental values becomes even more evident. The more rounded shape of the experimental loops in parallel geometry gives further support for distributed switching fields. These observations can be due to both distributed diameters/wall thicknesses of the NTs and distributed antiferromagnetic-like dipolar interactions among the NTs [58]. However, such interactions do not provide an increase of the average coercive field of bunched NTs, as directly proven by the simulations presented in Figure 7g. In conclusion, the theoretical simulations performed on the Ni NTs with realistic geometrical parameters lead to the coercive field values which are an order of magnitude lower than the experimental ones, suggesting the presence of an additional intrinsic anisotropy mechanism.

**Figure 8:**
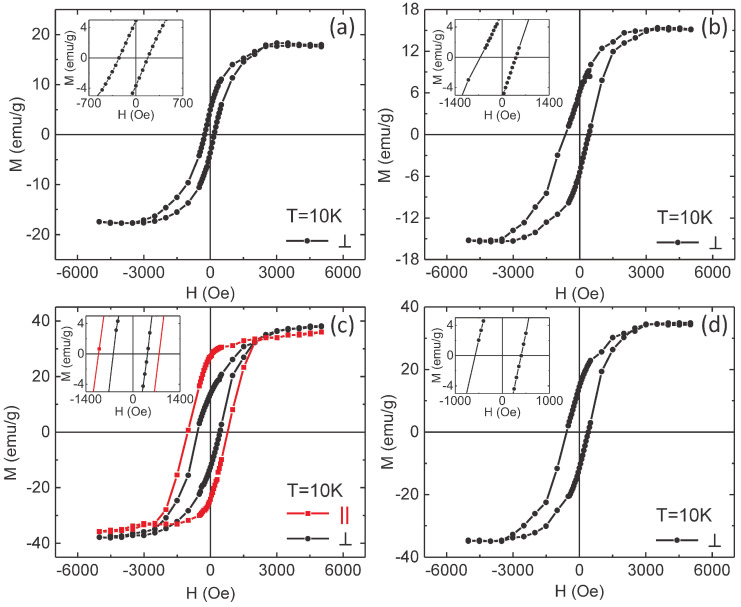
Hysteresis loops of samples S1 (**a**), S2 (**b**), S3 (**c**) and S4 (**d**), recorded at 10 K with the magnetic field applied perpendicular (black lines with dots) and parallel (red line with squares) to the NTs axis, after cooling the samples in a field of 2000 Oe from room temperature. The latter is shown only for S3. The insets represent corresponding closed-up magnifications around the coordinate system’s origin, showing the magnetic coercivity within the prepared samples.

On the other hand, open hysteresis loops in perpendicular geometry might be a consequence of misaligned NTs, but the eventually existing misaligned nanopores within the supported AAO template (used for the NTs confined growth) cannot lead to such dramatic changes, with loops in perpendicular geometry approaching the ones in parallel geometry. Therefore, other more relevant interactions have to respond also for the specific behavior of the experimentally obtained hysteresis loops in perpendicular geometry. A first hint for the additional anisotropy energy involved in these systems was provided by the hysteresis loops of the samples collected at 10 K and presented in Figure 8. It is worth noticing that these loops have been collected after cooling each sample in 2000 Oe from room temperature during the acquisition of the magnetization versus temperature curve. The striking peculiarities of the hysteresis loops presented in Figure 8 are the asymmetrical shapes in positive and negative fields observed mainly for sample S2 and the sensible negative shifts of the loops observed for all samples. This shift of the loop on the magnetic field axis, known as an exchange bias field, $H_{ex}$, together with an increased coercive field as compared to the case of a free F phase are the specific macroscopic traces of the unidirectional anisotropy induced on the F phase by the interfacial coupling to an AF phase. To note that the exchange bias field can be observed only below a critical temperature called the blocking temperature for the exchange bias effect ($T_{B}$), which is usually lower than the *Néel* temperature of the AF layer [14,17]. It is worth mentioning here that despite the similar name and notation, there is a conceptual difference between the blocking temperature for the exchange bias effect and the blocking temperature for a magnetic monodomain nanoparticle in the magnetic relaxation regime, as detailed in [59]. For the present systems, $T_{B}$ is lower than 300 K, where all the hysteresis loops are symmetrical on the magnetic field axis. Hence, the presence of the inner NiO AF layer pinning the concentric outer Ni layer is clearly demonstrated by the presence of the unidirectional anisotropy at the Ni/NiO interface. The effect can be particularly investigated in case of such hybrid uniaxial systems with cylindrical interfaces if the thickness of the two involved layers can be evaluated with reasonable accuracy.

**Figure 9:**
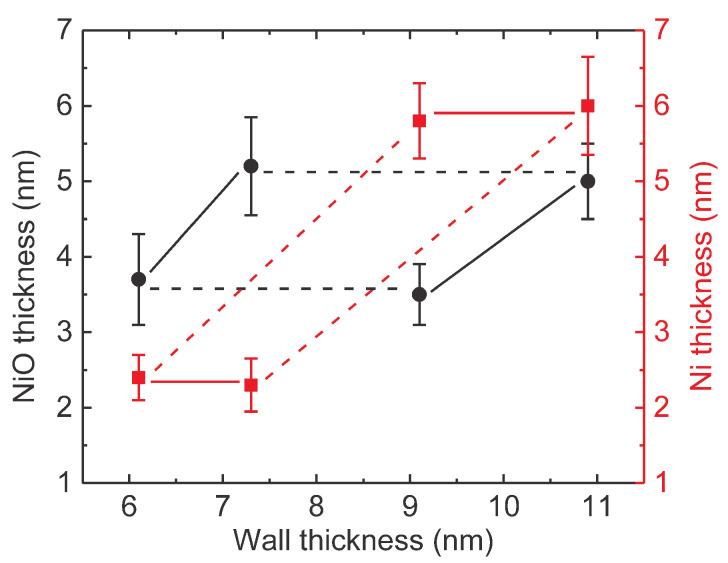
Thicknesses of the NiO layers (black dots) and Ni layers (red squares), as calculated from the differences in the saturation magnetization within the measured hysteresis loops. The solid lines show the cases when the $H_{ex}$ increases with antiferromagnetic (AF) layer thickness, while the dashed lines highlight the situation when $H_{ex}$ decreases with ferromagnetic (F) layer thickness. The overall wall thickness increases from S1 to S4.

Fortunately, the thickness of the Ni layer can be reasonably determined from: (i) the saturation magnetization values, assuming for the Ni layer a saturation magnetization typical for metallic Ni and for the NiO layer a null contribution, (ii) the area of each measured sample, (iii) the average length of the NTs, as well as (iv) the average center-to-center distance and average outer diameter for each sample, geometrical parameters acquired from the SEM analysis (see Section 3.1). The ratio between the observed saturation magnetization at 10 K and theoretical value for metallic Ni at low temperature (58.6 emu/g [49]) has been evaluated in order to approximate the quantity of Ni in the sample. The number of NTs in the sample was further estimated from the geometrical parameters mentioned above. The average amount of Ni per NT was then estimated and ultimately the average thickness of the Ni layer in the NT. Further, the thickness of the NiO layer was obtained by subtracting the as-determined thickness of the Ni layer from the overall wall thickness of the NTs. The graphical representation of the average wall thickness for each concentric component tube (Ni and NiO, respectively) for the analyzed samples (with increasing overall wall thickness from S1 to S4) is given in Figure 9. As can be observed, the obtained NTs can be grouped in 4 categories: (i) samples S1 and S2 with similar thickness of the Ni F layer (about 2.3(2) nm) but with different thicknesses of the NiO AF layer (3.6(3) and 5.1(3) nm, respectively), (ii) samples S3 and S4 again with the same thickness of the Ni F layer (about 6.0(3) nm) but with different thicknesses of the NiO AF layer (3.6(3) and 5.1(3) nm, respectively), (iii) samples S1 and S3 with similar thickness of the NiO AF layer (3.6(3) nm), but with different thicknesses of the Ni F layer (2.3(2) nm and 6.0(3) nm, respectively) and (iv) samples S2 and S4 with again the same thickness of the NiO AF layer (5.1(3) nm), but with different thicknesses of the Ni F layer (2.0(3) nm and 6.0(3) nm, respectively). The numbers in round brackets denote the reduced error range corresponding to the least significant digit of the NiO and Ni thicknesses, respectively.

**Figure 10:**
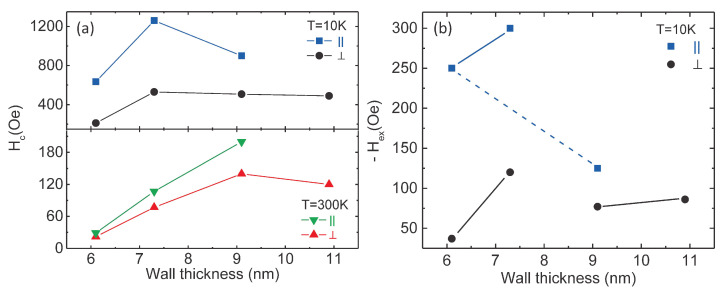
Magnetic parameters taken from Superconducting Quantum Interference Device (SQUID) measurements: (**a**) coercive fields determined at 300 K and 10 K in parallel and perpendicular geometry and (**b**) exchange bias fields of the analyzed samples at 10 K, where the solid lines show the cases when the $H_{ex}$ increases with AF layer thickness, while the dashed lines highlight the situation when $H_{ex}$ decreases with F layer thickness.

The specific parameters related to the unidirectional anisotropy effect ($H_{c}$ and $H_{ex}$) obtained from the hysteresis loops collected at 300 K and 10 K for all samples are shown in Figure 10. The first observation is related to the connection between $H_{ex}$ and the geometrical parameters for the F and AF interfaced concentric tubes. In case of planar interfaces (2D F/AF bilayers) earlier experimental results and theoretical models reported that in parallel geometry the exchange bias field is triggered by a critical thickness of the AF layer, afterward remaining constant with increasing the thickness of the pinning layer ($t_{AF}$), whereas it is inversely proportional to the thickness of the F layer, $H_{ex}\propto 1/t_{F}$ [14,17]. No specific dependencies in perpendicular geometry have been reported as far as we know.

Nevertheless, the present results show different characteristics of the exchange bias field versus the geometrical parameters of the concentric layers with F/AF cylindrical interfaces. First of all, by comparing samples S1 and S2, with the same thickness for the wall of the F tube, it can be observed that $H_{ex}$ increases with $t_{AF}$ in both geometries, anyway, in a more pronounced manner for the perpendicular geometry. However, $H_{ex}$ increases with $t_{AF}$ much less pronounced for an increased thickness of the F layer (see samples S3 and S4). On the other hand, in the case of parallel geometry, the exchange bias field decreases with the thickness of the F layer in a manner very close to the dependence $H_{ex}\propto 1/t_{F}$, as can be assumed for the equality of the products ($H_{ex}\cdot t_{F}$) in case of samples S1 and S3. In perpendicular geometry, such dependence is no longer respected, especially for a low thickness of the AF layer, where the exchange bias increases with $t_{AF}$ (see values of samples S1 and S3).

Concerning the coercive fields, they are almost one order of magnitude larger at 10 K as compared to 300 K and in addition, they respect closely the variation of the exchange bias field along the analyzed samples in both measuring geometries, proving definitely their direct connection to the unidirectional anisotropy induced at lower temperatures. At 300 K the coercive fields increase roughly with the overall wall thickness and also with the wall thickness of the Ni tube (see samples S1 and S3), in contrast to the theoretical estimations presented in Figure 7h. Hence, both the atypical behavior of the coercive field versus the wall thickness of a free F tube, as well as the much higher experimental values with respect to the theoretical ones, show that the unidirectional anisotropy influences the coercive field even at much higher temperatures than $T_{B}$. In addition, the morpho-structural properties of the samples might have also a sensible influence on the magnetic parameters of the NTs. For example, in the present case, a better structural quality of the NTs architectures together with a better crystallinity of individual NTs can be mentioned for the systems with a larger wall thickness of the NTs (obtained at more negative reduction potential) with direct influence on the uniaxial anisotropy constants, and finally on the coercive fields.

## 4. Conclusions

In this work, we demonstrated the simplicity and process feasibility to fabricate large dense arrays of vertically-aligned ferromagnetic Ni NTs with easily adjustable wall thickness. The method allowed further passive formation of ultra-thin antiferromagnetic NiO layers, to ultimately yield large forests of hybrid heterostructured F/AF tubular nanoarchitectures presenting unidirectional anisotropy. Using adequately adapted SQUID magnetic measurements, we demonstrated the ability to finely tune the magnetic behavior of the array just by simply changing the initial Ni NTs wall thickness through changing the cathodic reduction potential applied during the electrochemical growth of the nanostructures. Furthermore, it was shown that the ultra-thin passivation NiO layer is responsible for inducing unidirectional anisotropy at the Ni/NiO interface with direct implications on the magnetic properties of the pinned Ni NTs array. The magnetic results have been discussed with respect to additional micromagnetic simulations and the derived thicknesses of the interfaced concentric Ni and NiO layers. A step forward in understanding the connection between geometrical parameters and the exchange bias field as well as coercivity in 1D systems consisting of concentric F/AF layers with cylindrical interfaces has been done, while a critical discussion relative to 2D F/AF bilayers has been provided. Such dense arrays of pinned F NTs may potentially withstand individual magnetic addressability, by carefully tuning further their magnetic properties in terms of changing the magnetic packing fraction either by adjusting the template porosity, or ultimately by controlling the degree of NTs oxidation. The entire fabrication protocol assures full compatibility with the standard Si processing and is quite amiable to further optimizations, opening an outstanding pathway towards large-scale integrability and manufacturing of a new generation of magnetic devices relying on large dense arrays of vertically-standing quasi-1D nanostructures with easily tuneable magnetic features.

## Abbreviations

The following abbreviations are used in this manuscript:

NIMP	National Institute of Materials Physics
R&D	Research & Development
MDEO	R&D Center for Materials and Electronic & Optoelectronic Devices
IMCN	Institute of Condensed Matter and Nanosciences
UCLouvain	Université catholique de Louvain
IFIN-HH	Horia Hulubei National Institute for R&D in Physics and Nuclear Engineering
NWs	Nanowires
NTs	Nanotubes
PC	Polycarbonate
AAO	Anodic aluminum oxide
0D	Zero-dimensional
1D	One-dimensional
2D	Two-dimensional
F	Ferromagnetic
AF	Antiferromagnetic
GMR	Giant Magneto-Resistance
TMR	Tunneling Magneto-Resistance
DIW	Deionized water
DC	Direct current
SCE	Saturated calomel electrode
NHE	Normal hydrogen electrode
SEM	Scanning Electron Microscope
XRD	X-ray diffraction
SQUID	Superconducting Quantum Interference Device
RSO	Reciprocal Space Option
UEFISCDI	Executive Unit for Financing Higher Education, Research, Development and Innovation
FNRS	Fonds de la Recherche Scientifique
BSMA	Bio- and Soft Matter
